# TIDE: adjuvant tislelizumab plus donafenib combined with transarterial chemoembolization for high-risk hepatocellular carcinoma after surgery: protocol for a prospective, single-arm, phase II trial

**DOI:** 10.3389/fonc.2023.1138570

**Published:** 2023-04-17

**Authors:** Weili Qi, Wei Peng, Xin Qi, Zhancheng Qiu, Tianfu Wen, Chuan Li

**Affiliations:** ^1^ Department of Liver Surgery, West China Hospital, Sichuan University, Chengdu, China; ^2^ West China School of Medicine, Sichuan University, Chengdu, China; ^3^ Chinese Evidence-based Medicine Center, West China Hospital, Sichuan University, Chengdu, China

**Keywords:** hepatocellular carcinoma, adjuvant therapy, VEGF, PD-1, TACE

## Abstract

**Background:**

The high recurrence rate of hepatocellular carcinoma (HCC) after surgery negatively affects the prognosis of patients. There is currently no widely accepted adjuvant therapy strategy for patients with HCC. A clinical study of effective adjuvant therapy is still needed.

**Methods:**

In this prospective, single-arm, phase II clinical trial, an adjuvant regimen of donafenib plus tislelizumab combined with transarterial chemoembolization (TACE) will be used to treat enrolled HCC patients after surgery. Briefly, patients newly diagnosed with HCC by pathological examination who underwent curative resection and had a single tumor more than 5 cm in diameter with microvascular invasion as detected by pathological examination are eligible. The primary endpoint of the study is the recurrence-free survival (RFS) rate at 3 years, and secondary endpoints are the overall survival (OS) rate and the incidence of adverse events (AEs). The planned sample size, 32 patients, was calculated to permit the accumulation of sufficient RFS events in 3 years to achieve 90% power for the RFS primary endpoint.

**Discussion:**

Vascular endothelial growth factor (VEGF) and programmed cell death protein 1 (PD-1)/programmed cell death ligand 1 (PD-L1) pathways regulate the relevant immunosuppressive mechanisms of HCC recurrence. Our trial will evaluate the clinical benefit of adding donafenib plus tislelizumab to TACE in patients with early-stage HCC and a high risk of recurrence.

**Clinical trial registration:**

www.chictr.org.cn, identifier ChiCTR2200063003.

## Introduction

Hepatocellular carcinoma (HCC) remains one of the most common and fatal cancers worldwide, especially in China, where the annual number of new HCC cases and deaths associated with HCC reached 431,000 and 412,000, respectively (1). Surgical resection is still recommended as the main therapeutic strategy for early-stage HCC patients, but the overall prognosis of HCC patients after radical resection is poor due to the high recurrence rate (approximately 70% at 5 years after surgical resection) (2, 3). It is reasonable to believe that a reduction in the postoperative recurrence rate could help to improve the overall prognosis of HCC.

Although the current clinical guidelines do not recommend any adjuvant therapy, clinical investigators initiated some trials of adjuvant therapy for HCC after radical resection. It has been reported that interferon (IFN)-α can reduce recurrence and prolong survival time (4), but the conclusion remains controversial (5). The results of two randomized controlled trials (RCTs) from Wang et al. and Wei et al. showed that postoperative adjuvant transarterial chemoembolization (PA-TACE) therapy has the effect of reducing recurrence risk and prolonging survival (6, 7). However, inconsistent results were found in some other studies (8). Other studies also suggested that conventional TACE (c-TACE) suffers from unstable drug loading and inadequate drug dosing (9, 10). A meta-analysis of eight RCTs showed that cytokine-induced killer (CIK) cellular therapy reduced the postoperative recurrence rate at 1 year and 3 years and improved the overall survival rate from 1 to 5 years but had no effect on the 5-year recurrence rate and the 6-year overall survival rate (11). Several retrospective studies have shown that adjuvant therapy with sorafenib after hepatectomy is effective in preventing recurrence (12, 13). However, the STORM trial, the largest clinical trial of adjuvant therapy in HCC to date, failed to draw a solid conclusion regarding the use of sorafenib for adjuvant therapy for HCC (14). These results indicated that further exploration of adjuvant therapy for HCC is needed.

The LANCE study initiated by Qin et al. showed that patients in the lenvatinib plus TACE group had a significantly longer median recurrence-free survival (RFS) than those in the TACE only group (17.0 months *vs*. 9.0 months, P=0.02) (15). This undoubtedly enhanced the confidence in the combination of local and systemic adjuvant therapy to prevent the postoperative recurrence of HCC. At present, several clinical trials of adjuvant therapy for HCC after resection are in process, including nivolumab (CheckMate-9DX) or pembrolizumab (KEYNOTE-937), EMERALD-2 study and IMbrave 050 study. The regimens of these trials were previously investigated for patients with advanced HCC, and survival benefits were obtained.

In recent years, the advent of immune checkpoint inhibitors (ICIs) has transformed therapeutic strategies for various solid tumors. However, the effect of ICI only therapy was unsatisfactory (16, 17). Not only is the effective response rate to monotherapy low (18, 19), but there is insufficient knowledge of predictive biomarkers of ICI treatment response such as programmed death ligand 1 (PD-L1) expression, tumor mutational burden (TMB), and microsatellite instability (MSI) status (20, 21). Conversely, some studies have shown that antiangiogenic therapy can enhance the antitumor sensitivity of programmed cell death protein 1 (PD-1)/PD-L1 inhibitors, so antiangiogenic therapy combined with immunotherapy can achieve synergistic antitumor effects (22–24). The results of two multicenter phase III studies (Imbrave150 (25) and ORIENT32 (26)) showed that overall survival (OS) and progression-free survival (PFS) in the combination therapy group were significantly better than those in the monotherapy group. A systematic review and meta-analysis of 56 RCTs by Viscardi et al. showed that early death rates were significantly lower in patients treated with ICI plus other therapies than in patients treated with ICI therapy alone (6.7% *vs*. 14.2%) (27). Recently, we reported a novel conversion therapy strategy of TACE combined with tyrosine kinase inhibitor (TKI) and PD-1 inhibitor for patients with unresectable HCC at the 2022 American Society of Clinical Oncology Gastrointestinal (ASCO-GI) Cancers Symposium (28). Our statistical results showed that the objective response rate (ORR) was as high as 84.2%, the disease control rate (DCR) was as high as 94.7%, and the conversion resection rate was as high as 50%. Grade 3 adverse events (AEs) occurred in 22 patients (22/38, 57.9%), and no grade 4/5 AEs occurred. We initiated this phase II trial of TACE plus TKI and PD-1 inhibitor with the inspiration of LANCE, IMbrave 050 and EMERALD-2. Donafenib (TKI) and tislelizumab (PD-1 inhibitor) were selected for this trial.

Donafenib is a new generation of small molecule multikinase inhibitors. An open-label, randomized, parallel-controlled, multicenter phase II/III clinical study of donafenib as first-line therapy for advanced HCC (ZGDH3) included a total of 668 patients with advanced HCC (29). The results showed that the OS with donafenib was significantly longer than that with sorafenib (12.1 months *vs*. 10.3 months; P=0.0245). In addition, the AE spectrum of the two groups was similar, and the sorafenib group showed better tolerability than the sorafenib group. Donafenib is also the only monotherapy drug to date that has been superior to sorafenib in OS in a first-line head-to-head study in advanced HCC.

Tislelizumab is a humanized IgG4 monoclonal antibody against immune checkpoint inhibitory receptors with high affinity and binding specificity for PD-1. In a global multicenter, phase IA/IB clinical study (NCT02407990), tislelizumab was used as a second-line drug in the therapy of advanced HCC, and the results showed that 6 patients (6/50) achieved partial response (PR), and the ORR reached 12.2% (30). In another global single-arm, multicenter, open-label, phase II study (NCT03419897), the ORR by independent review committee (ORR IRC) of tislelizumab as a second-line drug for advanced HCC even reached 12.9%, exhibiting patient safety and good tolerance (31).

The purpose of this study is to observe and evaluate the efficacy and safety of the adjuvant therapy regimen of donafenib and tislelizumab combined with TACE in patients with a high risk of HCC recurrence after resection. We named this phase II, prospective, single-arm clinical trial as “TIDE”.

## Methods

### Study design

**Figure 1:**
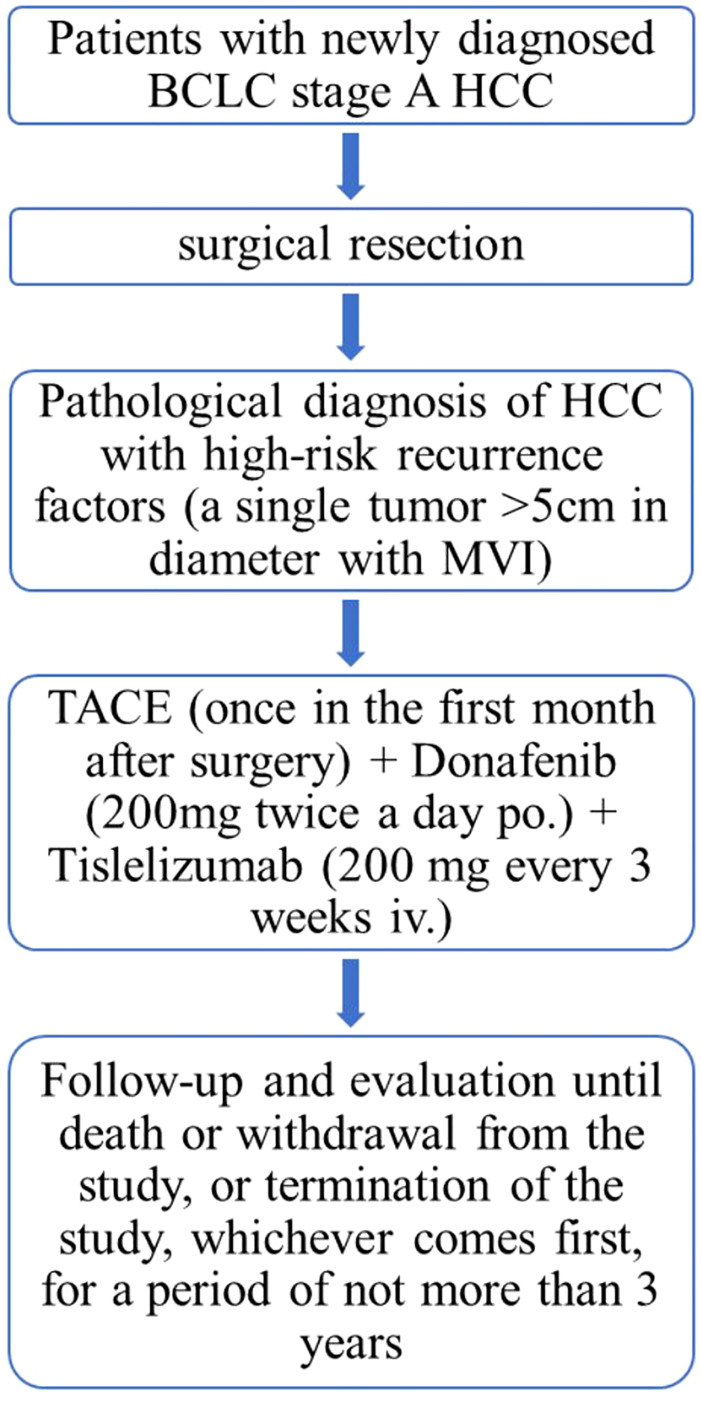
Flow chart of the study design.

This study is a single-center, single-arm, prospective, phase II trial to evaluate the efficacy and safety of donafenib plus tislelizumab combined with TACE in adjuvant therapy after surgical resection to delay HCC recurrence (**Figure 1**). The study will begin enrollment in September 2022, and approximately 32 patients who have undergone surgical resection and are at high risk for HCC recurrence will receive the abovementioned adjuvant therapy and will be followed up.

### Key eligibility criteria

#### Inclusion criteria

1) Voluntarily enrollment in the study and signed informed consent; 2) age 18~75 years old; 3) newly diagnosed HCC confirmed by pathological examination addressed by curative resection (R0 resection); 4) single tumor larger than 5 cm in diameter with microvascular invasion (MVI) and with or without satellite nodules; 5) no extrahepatic HCC; 6) complete recovery within 4 weeks after surgery; 7) Child Pugh A and ECOG performance status of 0 or 1; 8) adequate hematologic and vital organ function defined by laboratory test results; 9) for patients with hepatitis B virus (HBV) infection, HBV DNA <2000 IU/ml during screening, initiation of anti-HBV therapy at least 14 days prior to adjuvant therapy and willingness to continue anti-HBV. R0 resection was defined as follows: histological confirmation of surgical margin no residual tumor cells; decrease in alpha-fetoprotein (AFP) and des-gamma-carboxy-prothrombin (DCP) levels to normal levels at 1 month after surgery; and no evidence of residual lesions or recurrence on radiological evaluation at 1 month after surgery.

#### Exclusion criteria

1) Previous liver transplantation or presence on the waiting list for liver transplantation; 2) previous antitumor therapy, including local therapy, targeted therapy, immunotherapy, systemic chemotherapy, etc.; 3) any level of macrovascular invasion, including portal vein, hepatic vein, or vena cava invasion; 4) known fibrolamellar HCC, sarcomatoid HCC, or mixed hepatocellular-cholangiocellular tumor; 5) human immunodeficiency virus (HIV) infection autoimmune diseases; 6) known history of severe allergy to any monoclonal antibody or research drug excipients; 7) participation in other clinical studies, or the first dose is less than 30 days from the end of the previous clinical study drug therapy; 8) pregnant or breastfeeding status; 9) presence of other factors that may affect the results of the study or cause the study to be terminated halfway according to the judgment of the investigator; these include alcoholism, drug addiction, other serious diseases (including mental diseases) requiring combined therapy, serious abnormal laboratory tests, and family or social factors that affect drug safety.

#### Dropout case criteria

1) The investigator may decide to withdraw a patient from the study; 2) patients who have allergic reactions or serious AEs should cease participation in the trial according to the judgment of the investigator; 3) those who suffer from other complications during the trial and should not continue the trial; 4) patients with poor compliance may be excluded; 5) subjects can withdraw on their own.

### Study procedures

Eligible subjects will receive one cycle of TACE one month after surgery. Two weeks after TACE, the patient will be given tislelizumab (200 mg every 3 weeks iv.) and donafenib (200 mg twice a day po.) This regimen will be given for a period of 1 year unless disease recurrence or unacceptable toxicity occurs.

Subjects must undergo imaging examinations, including computed tomography (CT) of the chest, abdomen, and pelvis, within 28 days before the first dose. If there are relevant clinical indications, other body part scans and/or (18)F-fluorodeoxyglucose-positron emission tomography (FDG-PET)/CT imaging can be performed. Tumor assessments will be performed every 12 weeks (± 1 week) after the initiation of study therapy by investigators on contrast-enhanced CT or contrast-enhanced magnetic resonance imaging (MRI) per RECIST v1.1 or mRECIST, respectively (the number of assessments may increase based on clinical needs as determined by the investigators). Relevant safety assessments will be carried out 28 days ( ± 7 days) after the last medication and once every 24 weeks ( ± 7 days) after therapy, either by telephone or face-to-face visits, until the subject dies or withdraws from the study, or the study is terminated, whichever occurs first, for a duration of up to 3 years.

A separate case report form (CRF) will be created for each subject, and follow-up, registration, form filling and data maintenance will be performed by dedicated researchers.

### Sample size calculation

In a previous study on the effect of MVI on recurrence and prognosis after radical resection in 1517 patients with HCC, the 3-year RFS rate was less than 30% (32). We assumed that adjuvant therapy could increase the 3-year RFS rate to 50%. Using an alpha risk of 0.05 and a power of 80%, a sample size of 29 patients was needed, and considering a dropout rate of 10%, 32 patients needed to be enrolled.

### Study end points

The primary endpoint of the study will be 3-year RFS, defined as the time from surgery to the first documented disease recurrence. The secondary key endpoint is OS, which is defined as the time from randomization to death from any cause. Other secondary endpoints include the incidence of AEs, considering the number, severity, duration and outcome of AEs during therapy according to the National Cancer Institute Common Terminology Criteria for Adverse Events (NCI-CTCAE) v5.0 scale.

### Statistics

All analyses will be performed on an intention-to-treat (ITT) basis. The Kaplan−Meier method will be used to determine RFS and OS. Hazard ratios (HRs) and their 95% confidence intervals (CIs) will be estimated using Cox proportional hazards models. Statistical significance will be defined when the P value is less than 0.05. A safety monitoring subcommittee will be established to monitor patient safety and study progress. The safety analysis set will include all treated patients.

## Discussion

HCC is still an intractable disease. The high recurrence rate after surgical resection and the unsatisfactory overall prognosis have become major challenges for surgeons. Effective adjuvant therapy strategies are urgently needed to improve the OS and RFS of HCC patients. Currently, adjuvant therapy for HCC is still in the exploratory stage, and strategies for postoperative use of immunotherapy, targeted drugs, immunomodulators, hepatic arterial infusion chemotherapy (HAIC), and TACE alone or in combination are being actively carried out.

At present, it is believed that the high recurrence rate of HCC after resection is mainly related to the existence of microdisseminated foci or multisite tumors before surgery, especially in patients with high risk factors for recurrence (33). PA-TACE can supplement the treatment of occult residual lesions that cannot be detected before or during surgery, reducing the recurrence of HCC after radical hepatectomy, and it is well tolerated (6). Therefore, TACE is often recommended as an adjuvant therapy strategy for HCC patients with indicators of a high recurrence risk after surgical resection in China. Unfortunately, the results of TACE in HCC therapy remain unsatisfactory, and hypoxia secondary to TACE is thought to play a key role (34). Some studies suggest that hypoxia leads to the overexpression of hypoxia inducible factor-1α (HIF-1α), resulting in the upregulation of VEGF, which may induce tumor revascularization and local recurrence (35, 36). Therefore, combining TACE with a VEGF inhibitor can inhibit tumor vascular remodeling and tumor (re)proliferation. Moreover, TACE has been shown to enhance the immune response to ischemia-induced immunogenic cell death (ICD) and induce tumor-associated antigen-specific responses (37, 38). TACE has been reported to increase the number of CD4^+^ and CD8^+^ T cells in peripheral blood mononuclear cells (PBMCs) of patients, decreasing the number of regulatory T cells (Tregs) at the same time, but this immune response tends to be short-lived and may not provide long-term antitumor effects (39). TACE combined with ICI can further enhance the development of tumor-specific memory T cells and maintain the antitumor response of patients (39).

The tumor microenvironment (TME) in the liver is currently thought to play a key role in determining the prognosis of HCC. Angiogenesis and immune escape are considered hallmarks of cancer and are interdependent processes that often coexist and together contribute to tumorigenesis and progression (40). The expression levels of VEGF and PD-L1 are upregulated in most tumors and mediate tumor angiogenesis and immune escape (41, 42). Tumor angiogenesis is mainly regulated by VEGF, while VEGF and its receptors are considered to constitute one of the most effective signaling pathways during angiogenesis (43). Apart from its angiogenic roles, VEGF also mediates immunosuppression in the TME by driving tumor-associated immunosuppression factors, such as inducing vascular abnormalities, reducing tumor antigen presentation, inhibiting the proliferation and cytotoxic function of effector T cells, and increasing Tregs and myeloid-derived suppressor cells (MDSCs) (44). Moreover, PD-L1 is highly expressed on the surface of tumor cells, which combine with PD-1 to inhibit the function of effector T cells, reduce the proliferation of PD-1-positive cells, inhibit the secretion of cytokines, and induce apoptosis (45). Signaling through the PD-1/PD-L1 axis restricts T-cell interactions with dendritic cells (DCs) (46) and converts T helper (TH) cells with immunosurveillance functions to negative immunoregulatory Treg cells (47). Thus, inhibition of VEGF-induced signaling not only inhibits angiogenesis but also stimulates immune responses, whereas anti-PD-1 antibodies can cause antibody-dependent cell-mediated cytotoxicity and promote tumor vascular normalization (48, 49).

The limitations of this study come mainly from its single-arm design. First, the subjects in the experimental group and the external control group were difficult to compare because they were not from the same subject pool. Furthermore, the lack of a parallel control resulted in low internal validity.

In summary, TACE causes tumor hypoxia and necrosis, which has a positive regulatory effect on the immune microenvironment; immunotherapy enhances the antitumor immune response of T cells; antiangiogenic therapy normalizes the tumor vascular structure, promoting the tumor microenvironment from immunosuppressed to immunopermissive. Therefore, the combination of the three therapies has a potential synergistic effect.

## Data availability statement

The original contributions presented in the study are included in the article/supplementary material. Further inquiries can be directed to the corresponding author.

## Ethics statement

The ethics committee on Biomedical Research of West China Hospital of Sichuan University approved this study (IRB No. 2022-691). All participants will sign written informed consent prior to participation in any study activities.

## Author contributions

WP and CL proposed the study. WQ, WP, XQ, ZQ, and TW performed the research. WQ and WP wrote the first draft, and CL and TW reviewed the paper. All authors contributed to the interpretation of the study. All authors contributed to the article and approved the submitted version.
